# Eye movement characteristics and visual fatigue assessment of virtual reality games with different interaction modes

**DOI:** 10.3389/fnins.2023.1173127

**Published:** 2023-03-31

**Authors:** Lei Fan, Junjie Wang, Qi Li, Zhenhao Song, Jinhui Dong, Fangjun Bao, Xiaofei Wang

**Affiliations:** ^1^National Engineering Research Center of Ophthalmology and Optometry, Eye Hospital, Wenzhou Medical University, Wenzhou, China; ^2^School of Ophthalmology and Optometry and School of Biomedical Engineering, Wenzhou Medical University, Wenzhou, China; ^3^Key Laboratory for Biomechanics and Mechanobiology of Ministry of Education, Beijing Advanced Innovation Center for Biomedical Engineering, School of Biological Science and Medical Engineering, Beihang University, Beijing, China

**Keywords:** virtual reality, eye movements, visual fatigue, video games, interaction mode

## Abstract

This study aimed to investigate the eye movement characteristics and visual fatigue of virtual reality games with different interaction modes. Eye movement data were recorded using the built-in eye tracker of the VR device and eye movement parameters were calculated from the recorded raw data. The Visual Fatigue Scales and Simulator Sickness Questionnaire were used to subjectively assess visual fatigue and overall discomfort of the VR experience. Sixteen male and 17 female students were recruited for this study. Results showed that both the primary and 360 mode of VR could cause visual fatigue after 30 min of gameplay, with significant differences observed in eye movement behavior between the two modes. The primary mode was more likely to cause visual fatigue, as shown by objective measurements of blinking and pupil diameter. Fixation and saccade parameters also showed significant differences between the two modes, possibly due to the different interaction modes employed in the 360 mode. Further research is required to examine the effects of different content and interactive modes of VR on visual fatigue, as well as to develop more objective measures for assessing it.

## Introduction

Virtual reality (VR) is a technology that enables users to experience immersive, computer-generated environments. In recent years, VR has gained widespread popularity with a range of applications, including entertainment (DJSCOE, Vile – Parle (W), Mumbai et al., 2014), education (Ruan, 2022), and healthcare (Mirelman et al., 2011; Maples-Keller et al., 2017). The concept of the “metaverse,” a virtual shared space that is accessible through the internet, has also contributed to the promotion of VR. Additionally, the development of VR games has contributed to the growth of this technology, as gamers can experience a level of immersion that is impossible with traditional gaming platforms. As the application of VR continues to grow rapidly, it is crucial to understand its potential impact on eye health and explore quantitative measures for monitoring its effects.

Visual fatigue is a condition where the eyes become uncomfortable due to prolonged and intense focus on certain tasks. It can be caused by activities such as reading or staring at a digital screen for an extended period. It was defined as a decrease in the performance of the human visual system in specific conditions (Lynch and Hockey, 1984). VR can lead to uncomfortable symptoms such as eyestrain, dizziness, and other visual fatigue (Hua, 2017). The vergence-accommodation conflict (VAC) is the leading cause of visual fatigue in the VR (Iskander et al., 2019). The effects of VR on visual fatigue have been studied extensively using questionnaires. Previous studies have also demonstrated a strong correlation between eye movement and visual fatigue. Changes in eye movement speed, fixation duration, and blinking frequency reflect fatigue status and mental load (Kim et al., 2011a; Bang et al., 2014). Characteristics associated with blinking and eye movements can be used to assess visual fatigue. Wang and colleagues developed two machine learning models for assessing eye fatigue using features related to blinking and eye movements (Wang et al., 2019). By analyzing the relationship between VAC and variability in convergence angle, Iskander and colleagues proposed a visual fatigue likelihood metric based on biomechanical analysis of the oculomotor system (Iskander et al., 2019; Iskander and Hossny, 2021). Oculomotor behavior is also closely linked to the mechanical load on the eye and optic nerve tissues. Eye movement has been shown to cause a transient increase in intraocular pressure and deformation of the posterior eye globe (Wang et al., 2017). Excessive or abnormal eye movement may be a risk factor for glaucoma and myopia.

Most studies on the effects of VR on visual fatigue have focused on comparing the pre- and post-exposure state of the eye or comparing eye movement differences when viewing different video content (Mohamed Elias et al., 2019; Yoon et al., 2020). However, there has been limited research on the differences in visual fatigue and eye movement behaviors in different interactive modes within VR. Therefore, this study aims to investigate the visual fatigue differences and eye movement behaviors in different interaction modes of a VR game.

## Methods

### Subject recruitment and clinical examinations

In this study, 16 male and 17 female students were recruited from Beihang University. The mean age was 23.85 (standard deviation: 2.12) years. Exclusion criteria include abnormal stereo vision, strabismus, retinal disease, visual field defects or other eye diseases, limitations in body movement and balance, inability to understand and follow the instructions provided by the researchers, and inability to participate in the entire experimental process. Written informed consent was obtained from all participants. The study was reviewed and approved by the Biomedical Ethics Committee of Beihang University and was conducted in accordance with the guidelines of the Declaration of Helsinki.

### Virtual reality game and eye tracking

**Figure 1:**
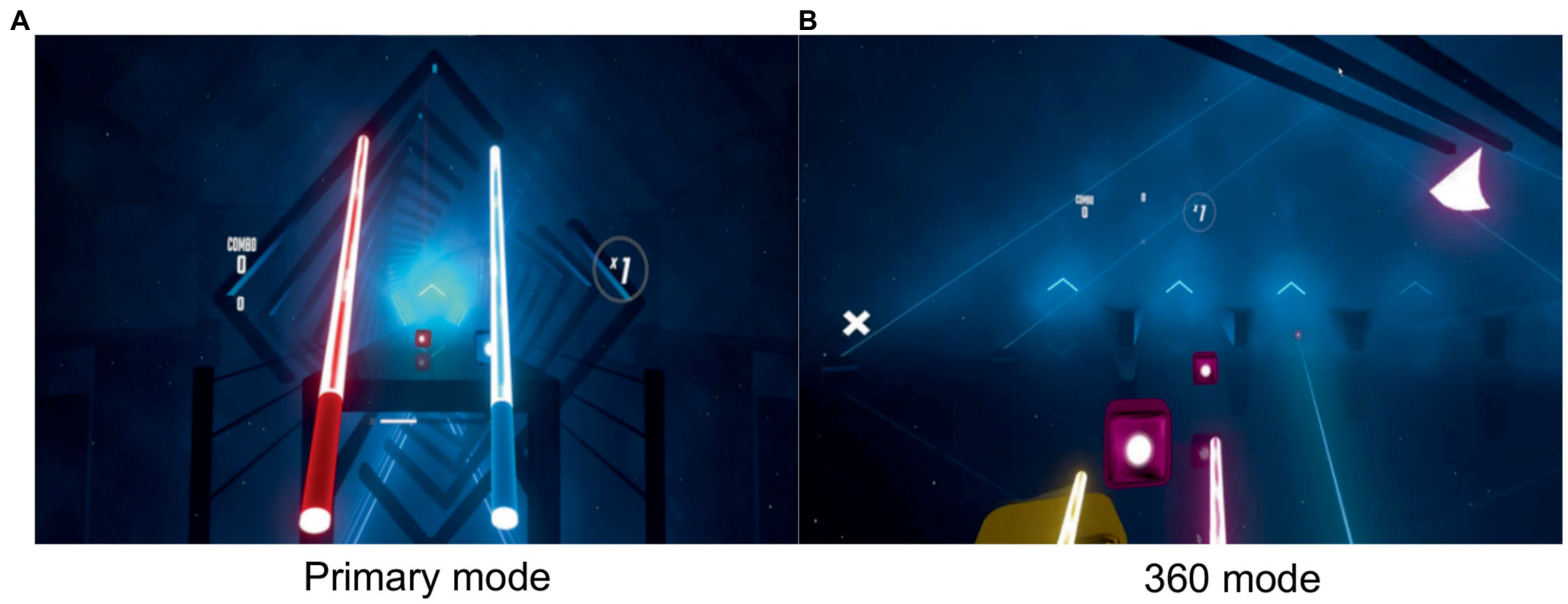
**(A)** In Beat Saber’s primary mode, a series of blocks is thrown at the player from directly ahead. It’s simple and straightforward, but can be very challenging at high levels. **(B)** In the 360 mode, notes can come from all around the player and making the game considerably more complex.

In the study, Virtual Reality games are used as visual stimuli, because games are considered to be the most attractive and immersive experience for users. It can enhance users’ reactions after games (Pallavicini et al., 2018). The game used in this study was Beat Saber (Beat Games). Beat Saber involves a substantial number of objects (fixation targets) that move toward the player’s eyes from a distance, necessitating continuous accommodation and convergence by the player’s eyes as the objects approach. These eye responses are closely linked to the development of visual fatigue. Furthermore, this game is widely popular and has been used in several studies as an experimental task to study symptoms of visual fatigue (Szpak et al., 2020; Banstola et al., 2022). Additionally, the game offers both primary and 360 modes, which perfectly aligns with the requirements of our study. This game simulates two controllers as lightsabers and objects (notes, bombs, obstacles, etc.) fly toward the player from a distance. Then the player must swing the lightsaber in their hands to slash the notes according to the music beat. Subjects were asked to play the game for 30 min in two interactive modes: primary mode and 360 mode. In the primary mode, the objects came from straight ahead of the player’s eyes, and the subject did not need to turn their head or body (Figure 1A). In the 360 mode, objects could come from around the player, requiring the player to turn their heads and bodies to look in different directions (Figure 1B).

The VR device used in this study was the HTC VIVE Pro Eye (HTC Corporation Inc.). It has a built-in eye tracker provided by Tobii (Tobii AB, Stockholm, Sweden). The eye tracker has a data output frequency of up to 120 Hz and an accuracy range of 0.5°–1.1°. In this study, the frequency of the eye tracker data was reduced to 30 Hz to minimize the computational load. A reliability study showed that 30 Hz was sufficient to accurately capture all the eye movement parameters used in this study, as compared to the 120 Hz data. We recorded the gaze direction at each time point, enabling us to capture eye movements in all directions within the raw data. Furthermore, the data for both eyes were simultaneously recorded, ensuring that all monocular information was included in the raw data. Raw data provided by the eye tracker includes the origin of the gaze, gaze vectors, eye openness, pupil diameter, and data validity. Raw data from the eye tracker was obtained using the HTC SRanipal SDK as .csv files, and custom Python code was used to calculate all the eye movement parameters.

### Subjective assessment of visual fatigue

Visual fatigue was subjectively assessed using the Visual Fatigue Scales consisted of 11 symptoms, each containing 5 identical symptom levels (0 = none, 1 = mild, 2 = moderate, 4 = severe; Wang and Cui, 2014). The scale is developed using the Delphi method, which is suitable for screening of visual fatigue in the general population, and each item is independent of others.

The overall discomfort of the VR experience was also assessed using a Simulator Sickness Questionnaire (SSQ). It includes 16 symptoms related to simulator disorders, which are divided into three subscales: nausea symptoms (N) such as hiccups, sweating, and increased salivation, etc.; oculomotor symptoms (O) such as eye strain, eye focus, blurred vision, etc.; disorientation problems (D) such as dizziness. Each symptom has 4 different levels of severity (0 = no symptoms, 1 = mild symptoms, 2 = moderate symptoms, 3 = severe symptoms). The total score is calculated by summing the weighted score of each item, with higher scores indicating more severe sickness symptoms.

### Experimental procedure

Before the experiment, general information such as gender and age was collected from the subject. All subjects had normal or best-corrected visual acuity of ≤0 LogMAR. All subjects were asked to observe a distance of more than 6 m for at least 30 min before the experiment to allow for eye relaxation. SSQ was then administered to assess the subjects’ condition before performing VR. Next, the subjects were randomly assigned to play the VR game in either the primary mode or the 360 mode. The SSQ was administered again after the game to assess the subjects’ condition. The second half of the experiment followed the same procedure as the first half, including observation at a distance of more than 6 m for at least 30 min, administering the SSQ, and playing the VR game in the other interactive mode. The SSQ was administered again after the game.

### Luminance of the VR game

Screen recordings of the VR game scene were obtained for all VR sessions. The luminance of the scene was calculated using the RGB intensity of all the pixels in each video frame, using the following formula: Luminance = 0.2126 × Red + 0.7152 × Green + 0.0722 × Blue (Nguyen and Brown, 2017). This method of calculating luminance is based on how the human eye perceives light and considers the different sensitivity of the three cone types in the retina to different wavelengths of light. The luminance values were used to evaluate the brightness of the VR game at each time point during the experiment.

### Data processing to obtain eye movement parameters

All data processing was conducted in Python (version 3.7.4, python.org). The fixation of the eye was determined using the I-DT algorithm (Blignaut, 2009). This algorithm requires the setting of two parameters: a dispersion threshold and a temporal threshold. In this study, these parameters were set to 0.02269 and 150 ms, respectively. After identifying fixations, several eye movement parameters were calculated: fixation duration, fixation angle, saccade duration, and saccade magnitude. Fixation duration was the time spent on a single fixation, and total fixation was the sum of all fixation durations. The average fixation duration was the mean of all fixation durations. Similarly, saccade duration and average saccade duration were calculated. The fixation angle was defined as the angle between the direction of a fixation point and the primary gaze direction. The saccade magnitude was defined as the angle between the vectors of two consecutive fixation points. The average and maximum saccade were also calculated. The average rotation angle of the eye was defined as the mean angle between all gaze vectors and the primary gaze direction. The blinking frequency and average blinking duration were then determined using the continuity and validity of the eye openness data. The device sampling rate is 30 Hz. Each row of data differs by about 33.3 ms. Judging invalid data of more than three consecutive rows as a single blink behavior. The average blink duration was ~100 ms.

### Statistical analysis

Statistical analyses were performed using Python (version 3.7.4, python.org). Data were expressed as mean ± standard deviation. The Kolmogorov–Smirnov test was used to assess the normality of the variables. Paired *t*-tests were used to compare differences in eye movement behavior between the two interactive modes of the VR game. Wilcoxon signed-rank tests were used to compare differences in subjective symptoms before and after VR in the two interactive modes. Pearson’s coefficient was used to analyze the correlation between scene luminance and pupil diameter. A *p*-value of <0.05 was considered statistically significant.

## Results

### Subjective assessment results

**Figure 2:**
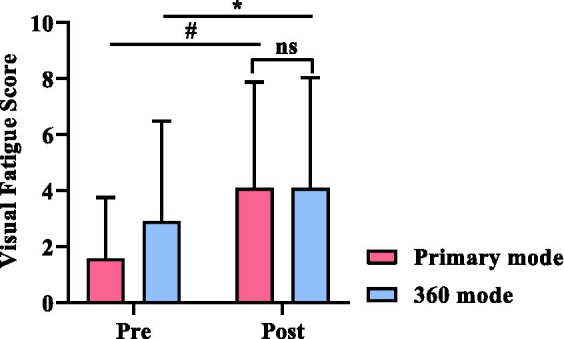
The changes in Visual Fatigue Score before and after the experiment. * indicates *p* < 0.05. ^#^ indicates *p* < 0.01.

The 360 mode and the primary mode showed a deepening of visual fatigue symptoms when compared before and after the experimental task (*p* < 0.05), but there was no difference between the visual fatigue scores when comparing the two scenes after the experiment (*p* = 0.712; Figure 2).

**Figure 3:**
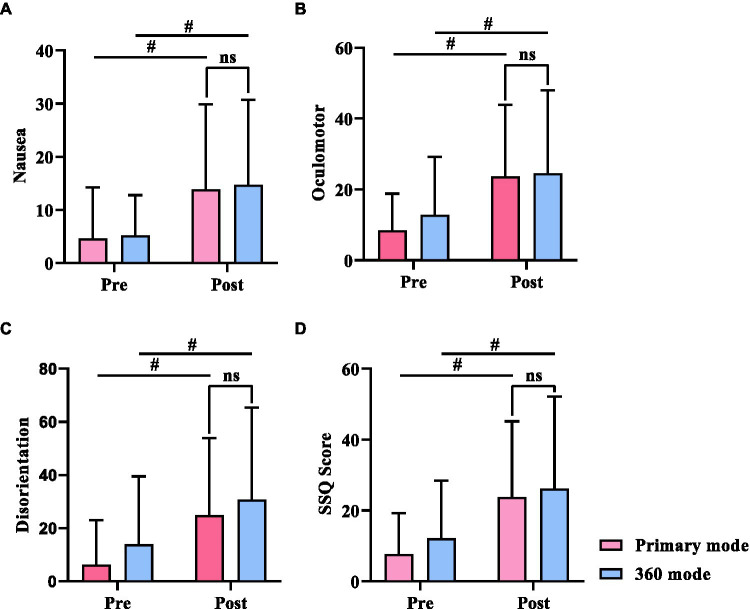
Three subscales and the score of Simulator Sickness Questionnaire (SSQ). **(A)** Nausea, **(B)** Oculomotor, **(C)** Disorientation, **(D)** SSQ score. * indicates *p* < 0.05. ^#^ indicates *p* < 0.01.

SSQ also showed a significant increase in all subscale scores when comparing the two scenes before and after the experiment (*p* < 0.01). As seen in Figure 3, the 360 mode produced greater discomfort symptoms than the primary mode, but there was no difference between the discomfort symptoms caused by the two (*p* > 0.05; Figure 3).

### Eye movement parameters

Table 1 shows the eye movement parameters in the primary mode and the 360 mode. The primary mode had higher fixation frequency, total fixation duration, and average fixation duration compared to the 360 mode (*p* < 0.05). There was no significant difference between the average fixation angle of both eyes. The 360 mode had higher total saccade duration, average saccade duration, average saccade magnitude of both eyes, and maximum saccade magnitude compared to the primary mode (*p* < 0.05). The 360 mode had a higher average blinking duration than primary mode (*p* < 0.05), but the blinking frequency was lower (*p* < 0.05). There was no significant difference between the average rotation angle of both eyes.

**Table 1:** Primary mode and 360 mode eye movement features analysis.

Eye movement parameters	Primary mode	360 mode	Value of *p*
Fixation frequency	110.60 ± 7.21	108.00 ± 6.26	**0.049**
Total fixation duration (s)	1003.59 ± 163.92	747.12 ± 110.74	**<0.001**
Average fixation duration (s)	305.48 ± 63.32	231.59 ± 38.58	**<0.001**
Average fixation angle: left eye (°)	12.70 ± 3.53	11.69 ± 3.10	0.074
Average fixation angle: right eye (°)	12.83 ± 3.59	11.92 ± 3.25	0.102
Total saccade duration (s)	788.13 ± 167.65	1049.09 ± 110.77	**<0.001**
Average saccade duration (s)	236.67 ± 43.68	324.67 ± 37.75	**<0.001**
Average saccade magnitude: left eye (°)	2.45 ± 0.48	3.07 ± 0.51	**<0.001**
Average saccade magnitude: right eye (°)	2.40 ± 0.42	3.02 ± 0.49	**<0.001**
Maximum saccade magnitude: left eye (°)	27.48 ± 4.32	30.35 ± 3.99	**0.004**
Maximum saccade magnitude: right eye (°)	27.46 ± 3.50	29.99 ± 3.57	**0.002**
Blinking frequency	20.05 ± 7.71	17.52 ± 7.26	**0.029**
Average blinking duration (s)	0.48 ± 0.34	0.67 ± 0.68	**0.035**
Average rotation angle: left eye (°)	12.70 ± 3.44	12.23 ± 2.95	0.396
Average rotation angle: right eye (°)	12.63 ± 3.53	12.30 ± 3.07	0.547

Values of indicators were shown as mean ± standard deviation (SD). The bold values indicate *p* < 0.05.

### Luminance of the VR game and pupil diameter

**Figure 4:**
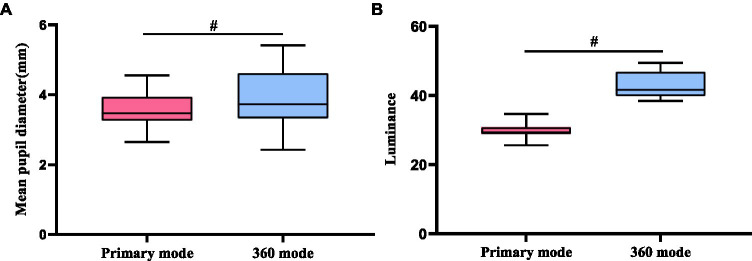
**(A)** Average pupil diameter of 360 mode vs. primary mode, **(B)** Brightness of 360 mode vs. primary mode. ^#^ indicates *p* < 0.01.

The pupil diameter in the 360 mode was larger than in the primary mode (Figure 4A). However, the luminance in the 360 mode was higher than in the primary mode (Figure 4B). Correlation analysis found no correlation between luminance and pupil diameter in either the 360 mode or the primary mode.

## Discussion

Eye movement behavior is closely related to visual fatigue and eye health. This study investigated the differences in eye movement behavior during different interaction modes in a virtual reality environment and assessed their impact on visual fatigue using subjective questionnaires. Results showed that both interaction modes of the VR experience could lead to visual fatigue, and there were significant differences in eye movement behavior between the primary mode and the 360 mode.

We used two questionnaires to evaluate the different symptoms induced by VR usage. The first questionnaire was the Visual Fatigue Questionnaire, which was used to assess only visual fatigue symptoms. The second questionnaire was SSQ, which was used to assess a range of physical discomfort symptoms induced by VR. Utilizing a combination of these two questionnaires could greatly enhance the strength of our study. The results showed that there were significant differences in visual fatigue scores (Figure 2) and SSQ scores (Figure 3) before and after the VR experience (*p* < 0.05). These findings suggest that subjects experienced a significant increase in visual fatigue and discomfort after playing VR games for 30 min in both primary and 360 modes. However, there were no significant differences in the scores of the questionnaires between the two modes in terms of visual fatigue or discomfort symptoms, including the three SSQ subscales of nausea, oculomotor, and disorientation symptoms.

It is important to acknowledge that using questionnaires to assess visual fatigue may have limitations due to varying perceptions and definitions of visual fatigue among individuals, as well as their applicability in certain contexts. Additionally, questionnaires may require a certain threshold of visual fatigue to be reached before they can detect it. Therefore, objective methods that can capture the physiological responses of the eye may increase the accuracy and sensitivity of the assessment. In this study, we incorporated eye movement parameters, including blinking and pupil size changes, to evaluate the state of the eye during VR gameplay. Numerous studies have investigated the relationship between eye movement parameters and visual fatigue (Kim et al., 2011a,b, 2018; Fourtassi et al., 2017; Wang et al., 2018, 2019; Parisay et al., 2020). Our findings indicated that there were notable differences in eye movement behavior between the primary mode and the 360 modes.

The measurement of blink parameters is a commonly used objective method for assessing visual fatigue (Heo et al., 2014), with a higher blinking frequency indicating a greater level of visual fatigue. Our results indicate that the blinking frequency is greater in the primary mode than in the 360 mode, whereas the average blinking duration is longer in the 360 mode than in the primary mode. These results suggest that the primary mode causes more visual fatigue than the 360 mode. In the primary mode, the fixation target is only presented from a fixed direction in front of the eyes, and participants are required to experience the virtual scene for 30 min, potentially resulting in boredom and fatigue. The increase in blinking frequency and decrease in blinking duration observed in this study may be a way of mitigating visual fatigue. Zhang and colleagues studied blink characteristics when viewing 3D and 2D videos and found that blinking frequency increased during 3D viewing and decreased during 2D viewing, with no significant differences in blinking duration between the two viewing modes (Zhang et al., 2013). Although our study is not directly comparable to theirs, both studies demonstrated differences in blink parameters when viewing the same content with different modes.

Pupil diameter is another common way of assessing visual fatigue. Previous studies have shown that a decreased pupil diameter is correlated with a more severe level of visual fatigue (Lee et al., 2013; Naeeri et al., 2021). In this study, the pupil diameter was significantly smaller in the primary mode than in the 360 mode (Figure 4A). It is important to note that differences in pupil diameter may also be affected by factors such as scene brightness (Cheng et al., 2006; Lee and Park, 2009) and mental load (Hess and Polt, 1964). However, in our study, the scene brightness was greater in the 360 mode than that in the primary mode (Figure 4B), and further correlation analysis revealed no significant correlation between luminance and pupil diameter in either mode. Thus, we can conclude that differences in pupil diameter between the two modes were not due to differences in scene brightness. The difference in pupil diameter between the two modes may be attributed to differences in task patterns. In the 360 mode, the areas of interest were distributed all around the subject, making the task more complex, exciting, and active, leading to a larger pupil diameter. Conversely, in the primary mode, the objects only come from the front of the subject, and participants may have experienced boredom, resulting in a smaller pupil diameter. These findings also align with our results for blink parameters, suggesting that the primary mode may be more likely to induce visual fatigue than the 360 mode.

Our results showed that the fixation frequency, average fixation duration and total fixation duration were greater in the primary mode than in the 360 mode (Table 1). However, there was no difference in the average fixation angle between the two modes. An increased fixation duration and decreased fixation frequency are a reflection of visual fatigue, as prolonged fixation time may indicate a need for more time to focus and process the information of interest (Naeeri et al., 2021). Feng et al. used fixation frequency and saccade amplitude to assess the effect of far-infrared therapy in relieving visual fatigue and demonstrated the effectiveness of these parameters in assessing visual fatigue (Feng et al., 2019). While there was no similar study performed before, previous studies can be used to interpret our results. Chapman and Underwood studied the driver’s eye movements while watching different scenes (Chapman and Underwood, 1998). They showed that, in rural areas with low visual complexity, eye movements showed longer fixation durations and shorter saccade distances, whereas in urban areas with high visual complexity, eye movements showed shorter fixation durations. In our experiments, the target in the primary mode comes only from a fixed direction in front of the eyes, allowing the participant to spend more time gazing at the target and processing the relevant information. In contrast, in the 360 mode, objects appeared from multiple directions, with a larger environmental area and more visual search required, resulting in shorter fixation durations. Taken together, our findings suggest that the fixation parameters indicate that the primary mode is more likely to cause visual fatigue than the 360 mode, which is consistent with the other parameters examined in our study.

Our study demonstrated a significant difference in saccade parameters between the primary mode and the 360 mode. Specifically, the total saccade duration, average saccade duration, saccade amplitude and maximum saccade amplitude of each eye were greater in the 360 mode than in the primary mode. These differences may be attributed to the interaction modes employed in the 360 mode, which has a larger play area and visual stimulus targets from all around the subjects. This finding is similar to a study on driving on different roads, which found that saccade lengths were longer for urban clips than rural clips (Chapman and Underwood, 1998). Urban roads are generally more complex and hazardous, resulting in greater eye movement behavior, with the human eye requiring constant reception and processing of information.

Our study has several limitations that warrant further study. Subjective measures of visual fatigue are not always accurate, especially when conducting brief virtual reality immersion experiments. During our experiment, participants were standing for the entire 30-min duration. However, in VR, subjects are often more excited and the resulting visual fatigue may be less pronounced and more difficult to assess subjectively. For future studies, it is recommended to extend the experimental time and identify more suitable experimental tasks for assessing visual fatigue in VR environments.

## Conclusion

Our study found that both the primary and 360 modes of VR could cause visual fatigue after 30 min of gameplay, with significant differences observed in eye movement behavior between the two modes. Results from objective measurements of blinking and pupil diameter suggest that the primary mode is more likely to cause visual fatigue. Fixation parameters and saccade parameters also showed significant differences between the two modes, possibly due to the different interaction modes employed in the 360 mode. Further study is needed to fully understand the effects of VR on visual fatigue and develop strategies for mitigating any negative effects. This includes researching the impact of various VR content and interactive modes on visual fatigue and developing more objective methods for assessing it.

## Data availability statement

The data analyzed in this study is subject to the following licenses/restrictions: The data that support the findings of this study are available at reasonable request from the corresponding author. The data are not publicly available as it is part of an ongoing larger study. Requests to access these datasets should be directed to XW, xiaofei.wang@buaa.edu.cn.

## Ethics statement

The studies involving human participants were reviewed and approved by Biomedical Ethics Committee of Beihang University. The patients/participants provided their written informed consent to participate in this study.

## Author contributions

LF and JW contributed equally to this work and both are first authors. LF, JW, and XW contributed to the conception of the manuscript, as well as to its writing and revision. LF, ZS, JD, and QL were responsible for collecting and analyzing the data. JW, FB, and XW provided research guidance and supervision throughout the project. All authors contributed to reading and approving the submitted version.

## Funding

This research was supported by National Natural Science Foundation of China (12272030), the 111 Project (B13003) and the Fundamental Research Funds for the Central Universities.

## Conflict of interest

The authors declare that the research was conducted in the absence of any commercial or financial relationships that could be construed as a potential conflict of interest.

## Publisher’s note

All claims expressed in this article are solely those of the authors and do not necessarily represent those of their affiliated organizations, or those of the publisher, the editors and the reviewers. Any product that may be evaluated in this article, or claim that may be made by its manufacturer, is not guaranteed or endorsed by the publisher.
